# Time-domain NIRS system based on supercontinuum light source and multi-wavelength detection: validation for tissue oxygenation studies

**DOI:** 10.1364/BOE.431301

**Published:** 2021-09-30

**Authors:** Aleh Sudakou, Frédéric Lange, Helene Isler, Pranav Lanka, Stanislaw Wojtkiewicz, Piotr Sawosz, Daniel Ostojic, Martin Wolf, Antonio Pifferi, Ilias Tachtsidis, Adam Liebert, Anna Gerega

**Affiliations:** 1 Nalecz Institute of Biocybernetics and Biomedical Engineering, Warsaw, Poland; 2 Department of Medical Physics and Biomedical Engineering, University College London, London, UK; 3 Department of Neonatology, University Hospital Zurich, University of Zurich, Zurich, Switzerland; 4 Dipartimento di Fisica, Politecnico di Milano, Milano, Italy

## Abstract

We present and validate a multi-wavelength time-domain near-infrared spectroscopy (TD-NIRS) system that avoids switching wavelengths and instead exploits the full capability of a supercontinuum light source by emitting and acquiring signals for the whole chosen range of wavelengths. The system was designed for muscle and brain oxygenation monitoring in a clinical environment. A pulsed supercontinuum laser emits broadband light and each of two detection modules acquires the distributions of times of flight of photons (DTOFs) for 16 spectral channels (used width 12.5 nm / channel), providing a total of 32 DTOFs at up to 3 Hz. Two emitting fibers and two detection fiber bundles allow simultaneous measurements at two positions on the tissue or at two source-detector separations. Three established protocols (BIP, MEDPHOT, and nEUROPt) were used to quantitatively assess the system’s performance, including linearity, coupling, accuracy, and depth sensitivity. Measurements were performed on 32 homogeneous phantoms and two inhomogeneous phantoms (solid and liquid). Furthermore, measurements on two blood-lipid phantoms with a varied amount of blood and Intralipid provide the strongest validation for accurate tissue oximetry. The retrieved hemoglobin concentrations and oxygen saturation match well with the reference values that were obtained using a commercially available NIRS system (OxiplexTS) and a blood gas analyzer (ABL90 FLEX), except a discrepancy occurs for the lowest amount of Intralipid. *In-vivo* measurements on the forearm of three healthy volunteers during arterial (250 mmHg) and venous (60 mmHg) cuff occlusions provide an example of tissue monitoring during the expected hemodynamic changes that follow previously well-described physiologies. All results, including quantitative parameters, can be compared to other systems that report similar tests. Overall, the presented TD-NIRS system has an exemplary performance evaluated with state-of-the-art performance assessment methods.

## 1. Introduction

Time-domain near-infrared spectroscopy (TD-NIRS) uses short pulses of light to non-invasively and in real-time monitor tissue optical properties, i.e. absorption (*µ*_a_) and reduced scattering (*µ′*_s_) coefficients, which are related to the concentrations of chromophores in tissue, e.g. oxyhemoglobin (HbO_2_) and deoxyhemoglobin (Hb). TD systems acquire the highest amount of information, for a defined number of source-detector pairs, compared to continuous-wave and frequency-domain acquisition modes [1]. The monitored parameters, e.g. cerebral metabolism [2] (related to changes of chromophores concentrations) or tissue oxygenation [3] (related to absolute values), help diagnose physiological conditions and monitor results of clinical interventions. NIRS is safe and non-ionizing, which makes it suitable for studies on neonates [4–6] and on adults [7].

A number of recent publications review instrumental developments for various applications of NIRS, including the past and the current status of TD-NIRS [8], clinical brain monitoring with TD-NIRS [1], metabolic brain measurements on neonates [9], neuromonitoring for neonatal encephalopathy [10], functional NIRS [7], instrumentation for functional NIRS [11], and applications related to stroke patients [12], studies on muscles [3], and evaluating the level of consciousness typically after a brain injury [13]. Continuous advancements in optical technologies and electronics for TD systems are leading to better measurements and a wider range of applications. The advancements have enabled TD-NIRS measurements at larger source-detector separations, e.g. 9 cm [14], at longer wavelengths, e.g. in the range from 600 to 1350 nm [15], with more sources and detectors, e.g. 16 sources and 8 detectors with fibers [16], or 1032 detectors [17], or 36288 detectors [18], with fast acquisitions, e.g. wavelength switching at 160 Hz [19], more compact, e.g. 200 × 160 × 50 mm^3^ [20], wearable [21,22] and also wireless [23], or with an improved light harvesting capability that allows collecting more photons than restricted by the pile-up limit [24]. Another direction of advancements involves integrating various imaging techniques, e.g. TD-NIRS and diffuse correlation spectroscopy [25,26].

Measurements at more wavelengths allow estimating absolute concentrations of more chromophores, e.g. lipid, water, and collagen [15], in addition to HbO_2_ and Hb. Furthermore, to follow dynamic changes associated with physiology requires fast acquisitions on a time scale of typically 1 s. Of particular interest is monitoring concentration changes of cytochrome-c-oxidase (CCO) enzyme, which requires many wavelengths as well as fast acquisitions [2]. The importance of monitoring changes in CCO, which relates to tissue metabolism, and the relevant advances in optical technologies were reviewed in [9]. Recently, Lange *et al*. [19] presented an assessment study of a multi-wavelength TD-NIRS system that was designed for monitoring changes in CCO in addition to HbO_2_ and Hb. The system uses a broadband light source coupled with two acousto-optic tunable filters that allow switching between many different wavelengths. Other common approaches for multi-wavelength TD-NIRS are time multiplexing [27] and space multiplexing [16]. In the first approach, multiple pulsed lasers with different wavelengths are shined in parallel and the optical pulses of different lasers are delayed relative to each other such that they arrive with different time offsets to a single TCSPC card and get recorded in the same TAC, which allows a limited number of wavelengths, typically two. In the second approach, multiple pulsed lasers are sequentially shined using an optical switch, which allows employing many wavelengths at the cost of acquisition time. Renna *et al.* [27] presented such system with 8 pulsed diode lasers, but recommend sequential scanning of no more than 2 or 3 wavelengths for monitoring dynamic processes.

In the present work, the multi-wavelength TD-NIRS system avoids switching wavelengths. The principle of this kind of instrument was introduced in [28]; the presented system is an advanced version and with modern electronics. We report the most relevant performance tests from three established protocols for photon migration instruments (MEDPHOT [29], BIP [30], and nEUROPt [31]), which were intended for quantitatively assessing the performance of time-domain instruments that use pulsed laser sources, single-photon detectors, and time-correlated single-photon counting electronics. Measurements on 32 homogeneous phantoms and a solid inhomogeneous phantom [32] were carried out as part of the BitMap campaign [33], which is a multi-laboratory effort to standardize the performance assessment of instruments for diffuse optics. Furthermore, the ability to estimate absolute concentrations of HbO_2_ and Hb, and hence oxygen saturation, is assessed on a series of blood-lipid phantom measurements using the phantom and the protocol developed by Kleiser *et al.* [34,35]. Lastly, *in-vivo* monitoring during hemodynamic changes is demonstrated using measurements on the forearm of three healthy volunteers during arterial and venous cuff occlusions. This study is intended to quantify the capabilities of the system and support its clinical use for diverse applications.

## 2. System description

### 2.1 Overview

The system measures the distributions of times of flight of photons (DTOFs) in parallel for 16 wavelengths with a sampling time of 0.3 s. Two emission fibers allow delivering light at two locations. Two detection fibre bundles allow measuring at up to two source-detector distances (ρ). Therefore, the system provides 32 DTOFs (16 wavelengths and 2 detection modules) at a sampling rate of up to 3 Hz. A previous version of the system was used for hemodynamic studies involving the injection of an optical contrast agent Indocyanine Green [36,37]. The system was designed for the clinical environment with the possibility to continuously monitor patients before, during, and after surgery. The current fiber holder design is suitable for adults and can be potentially adjusted for neonates.

### 2.2 Housing setup

**Fig. 1.**
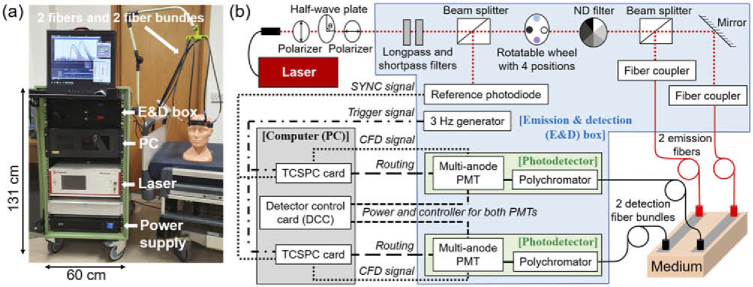
(a) Photo of the system. (b) Schematic diagram of the components’ layout. The acronyms are: emission and detection (E&D) box, neutral density (ND) filter, multi-anode photomultiplier tube (PMT), and time-correlated single-photon counting (TCSPC) card.

A photo of the arrangement of the system is shown in Fig. 1 (a) and a schematic diagram of the inner components is shown in Fig. 1 (b). All components were mounted inside a 19-inch metal rack case with four wheels, which is a commonly used housing setup for TD-NIRS systems because it is compact, portable, and robust [19,38]. An uninterruptible power supply unit (APC Smart UPS SMC1500I-2U) protects against interruption of external power supply and also allows the system to operate without an external power supply for a brief period.

### 2.3 Emission module

The results in this study were obtained with fiber laser SC480-8 WhiteLase (Fianium, UK), which operates at 80 MHz repetition frequency. The fundamental pulse width is 6 ps, the maximum output power is up to 8 W, and the spectral bandwidth range is from 460 to 2000nm.

(1) The output beam of the laser first passes through a system of two polarizers with a half-wave plate in-between, which serves as a variable attenuator for unpolarized light. For a chosen output power of the laser, the half-wave plate is manually rotated and then secured with a screw, such that the power of light exiting any of the two emission fibers is always lower than 20 mW, which equates to a power density of less than 2 mW/mm^2^ on the surface of the skin due to angular spread and distance, which is in line with the safety regulations for measurements on patients. Next, the beam passes through two edgepass filters, which remove the wavelengths that are outside of a desired spectral range. The current chosen spectral range is from 650 to 850 nm, which is achieved with a longpass filter (FEL0650 with a cut-on wavelength of 650 nm, Thorlabs, Sweden) and a shortpass filter (FES0850 with a cut-off wavelength of 850 nm, Thorlabs, Sweden). The two filters allow some unwanted light above 1200 nm to be emitted, but this light does not pass through the polychromator on the detection side.
(2) A beam splitter directs a fraction of the laser light to a reference photodiode (PHD 400, Becker & Hickl GmbH, Germany), which feeds a signal into the SYNC input of each TCSPC card. The details of using reference photodiodes can be found in the TCSPC handbook, pages 76 and 202 [39].
(3) The rest of the light passes through one of four openings in a rotatable wheel: i) an unimpeded opening that lets all light pass through, ii) a solid wall that blocks all light, iii) a band-pass filter centered around 780 nm (FL780-10, Thorlabs), which is used to calibrate diffraction gratings of the two polychromators, and iv) a bandpass interference filter (750 nm with FWHM of 50 nm, Edmund Optics), which is used for the excitation of the ICG in the fluorescence mode measurements.
(4) Final filtering is achieved with the use of a continuously variable neutral density filter (NDC-50C-2-B, Thorlabs), which removes a variable amount of excessive light power.
(5) A beam splitter directs the filtered light into two fiber couplers that guide the light into two optical fibers, which deliver the light to the surface of a medium, e.g. tissue or phantom, at two chosen locations. The parameters of each emission fiber are: step index, 0.39 NA, 2 m length, and 400 μm diameter (FT400EMT, Thorlabs).

### 2.4 Detection module

After travelling through a diffusive medium, the remitted light is collected by two separate detection fiber bundles (2 m length, 0.22 NA, and a 90° bent tip on the patient side with a 30 mm long-term bending radius, custom-made, Ceram Optec), which are terminated with a circular tip (diameter 3.6 mm) on the medium side and a line-shaped tip (1.4 × 7.3 mm) that fits into the slit of a polychromator; illustrated in the TCSPC handbook, page 171 [39].

Each fiber bundle guides light to one of the two separate detection modules (PML Spec, Becker & Hickl GmbH, Germany). Each detection module is comprised of two parts: a polychromator (MS125, Oriel Instruments, USA), which has 0.135 NA and uses 77414 diffraction grating (Grating Groove Density is 600 lines / mm), and a multi-anode photomultiplier tube detector (PML-16-1-C, Becker & Hickl GmbH, Germany). The power to both photomultiplier tubes is provided by a detector control card (DCC-100, Becker & Hickl GmbH, Germany), which also allows controlling gain parameters and overload shutdown. The width of each spectral channel is about 12.5 nm, which can be changed by using another diffraction grating, e.g. 35, 18, 9, or 4.5 nm as in [28]. The spectral range, which is a parameter of a polychromator, was set from 674 to 874 nm. Consequently, the central wavelengths of spectral channels were: 680, 692.5, 705, 717.5, 730, 742.5, 755, 767.5, 780.5, 792.5, 805, 817.5, 830, 842.5, 855, and 867.5 nm. For a further description of the detection module, we refer to the instrumentation section in the previous version of the system [36].

The output electrical signals of two PML-16-1-C are passed to two 16-channel TCSPC cards (SPC 150, Becker & Hickl GmbH, Germany). The synchronization between the acquisitions of the two TCSPC cards was achieved using the PCI Express I/O card (PCIe-6321, National Instruments), which was configured with LabView software (National Instruments). The card generates a trigger signal at 3 Hz for the start of the recording of the DTOF. The two TCSPC cards were used in continuous flow mode with a collection time of 0.3 s. During data analyses, the collection time can be increased by summing consecutive measured DTOFs. The parameters of a TCSPC card were configured such that each DTOF contained 1024 time bins with about 9.77 ps width each. For typical measurements, a temporal resolution of a recorded DTOF must be on the order of 10 ps to quantify the optical properties of diffuse media with accuracy better than 5% using a curve fitting method [40].

### 2.5 Software

All components were operated using the software provided by the manufacturers and run on a PC with Windows 7 Professional. The laser functions were controlled using the Fianium Laser Controller software. The DCC card and the two TCSPC cards were operated using the TCSPC Package 6.5.0 software (Becker & Hickl GmbH, Germany), which allows to adjust all TCSPC-related parameters, record the data, and assess the quality of the data before, during, and after a measurement. All data were saved and later processed offline using custom codes written in MatLab R2020b.

### 2.6 Optode holder

The two emission fibers and the two detection fiber bundles can be attached to the surface using custom 3D printed optode holders with holes at desired ρ (typically a few cm apart). The chosen printing materials were pure black ABS filament (Zortrax) for phantom measurements and black Fiberflex 40D filament (Fiberlogy) for *in-vivo* measurements, which is a more flexible material and achieves better contact with the skin. For phantom measurements, the optode holder was held in place using a support stand and covered with tape. For *in-vivo* measurements, the optode holder was fixed in place using Velcro strips and covered with a cohesive bandage.

## 3. Methods: measurements and data analysis

### 3.1 BIP protocol

The basic instrument protocol (BIP) [30] allows the assessment of parameters that are related to the hardware components of a time-domain instrument. In particular, we report the instrument response function (IRF), the differential nonlinearity (DNL), the warm-up, and the temporal fluctuations.

To measure the IRF [41], an emission fiber was aligned with a detection fiber bundle at a distance of 6 cm with a neutral density filter between them and two pieces of white paper covering the fiber and the fiber bundle. The DNL was assessed by recording the response to a battery-operated light-emitting diode, which produced a continuous light signal. Repeated measurements were summed to obtain more than 10^5^ photon counts per time bin for a good signal-to-noise ratio. The deviation in the number of photons detected in different time bins was quantified by calculating the peak-to-peak difference normalized to the mean value (ε_DNL_) [30]. To assess the warm-up, the IRF was repeatedly acquired for up to 6 hours. Afterward, to assess the temporal fluctuations, the DTOF was repeatedly acquired on a homogeneous solid phantom (label B2 in the MEDPHOT protocol) at ρ = 3 cm. The first three statistical moments, i.e. the total number of photons (*N*), the mean time of flight (*m*_1_), and the variance (*V*) [31], were calculated after background subtraction and using time bins that have photon counts higher than 1% of the maximum. Each DTOF contained about 10^6^ photons, which was achieved by integrating repeated acquisitions. The temporal fluctuations of the moments were assessed using the standard deviation of 100 values.

### 3.2 MEDPHOT protocol

The MEDPHOT protocol [29] allows the assessment of a system’s capability to recover *µ*_a_ and *µ′*_s_ in terms of linearity, coupling, and accuracy. The protocol includes measuring on 32 solid homogeneous phantoms that are labeled with a letter and a number. The phantoms make up all combinations of four nominal *µ′*_s_ values and eight nominal *µ*_a_ values (see the caption of Fig. 3). The base component of these phantoms is epoxy resin and we assumed the refractive index as 1.55. A measurement on each phantom was acquired for 30 s.

Four scatter plots were obtained: the measured *µ*_a_ (or *µ′*_s_) versus the nominal *µ*_a_ (or *µ′*_s_) and the measured *µ*_a_ (or *µ′*_s_) versus the nominal *µ′*_s_ (or *µ*_a_). Using the first two plots, the mean deviation in *µ*_a_ (or *µ′*_s_) from linearity was quantified by fitting a straight line and calculating the mean percentage difference between the measured data and the fitted line. This calculation does not use the values of nominal optical properties. Using the other two plots, the coupling of Δ*µ*_a_ to Δ*µ′*_s_ (or Δ*µ′*_s_ to Δ*µ*_a_) was quantified by fitting a straight line and calculating the slope, which requires using the changes of nominal values (assumed 0.05 cm^-1^ for Δ*µ*_a_ and 5 cm^-1^ for Δ*µ′*_s_).

The nominal values of *µ*_a_ and *µ′*_s_ of the 32 phantoms do not match the true values, and there is no established consensus yet on the agreed true values, as pointed out in [24]. Also, *µ*_a_ and *µ′*_s_ depend on wavelength, especially *µ′*_s_ [27]. Therefore, to assess the accuracy, we present the obtained spectra of *µ*_a_ and *µ′*_s_ for three well-characterized phantoms: phantom B2, the solid inhomogeneous phantom, and the 1% aqueous solution of Intralipid (the two inhomogeneous phantoms are presented in the next section). The obtained values can be compared to the reported results of other systems that measured on the same phantoms at multiple wavelengths.

### 3.3 nEUROPt protocol

The nEUROPt protocol [31] allows the assessment of a system’s sensitivity to a small localized *µ*_a_ perturbation positioned at various depths and later positions. Measurements were repeated on two inhomogeneous phantoms (solid and liquid) that had similar *µ′*_s_ but different *µ*_a_. Monte Carlo simulations were performed for a qualitative comparison.

The mechanically switchable solid inhomogeneous phantom was described in detail in [32]. The phantom has a cavity (diameter 14 mm) for a movable cylindrical rod that has a localized cylindrical *µ*_a_ perturbation (we used the rod with the following perturbation: 5 mm in diameter and length, i.e. 98 mm^3^ volume, and Δ*µ*_a_ equivalent to 0.017 cm^-1^). The rod can be precisely moved by a step motor.

The liquid phantom was a 1% aqueous solution of Intralipid (Fresenius Kabi AG, Bad Homburg, Germany) poured inside a dedicated cell [42]. A localized *µ*_a_ perturbation was introduced using a submerged small black PVC cylinder (4 mm in diameter and length, i.e. 50 mm^3^ volume), which was fixed to an end of a 0.5 mm thin, rigid, white metallic wire, which was verified to have a negligible effect on TD-NIRS measurements [42]. The depth and the lateral position of the metallic wire can be manually changed with a precision of about 1 mm.

The reported optical properties of the two phantoms without a perturbation are: *µ*_a_ ∼0.1 cm^-1^ (∼0.026 cm^-1^) and *µ′*_s_ ∼8 cm^-1^ (∼10.5 cm^-1^) at 800 nm for the solid [32] (liquid [19,43]). The solid phantom mimics the typical *µ*_a_ and *µ′*_s_ of biological tissue. The liquid phantom, which contained only water and Intralipid and hence can be easily replicated, is used for a comparison with the results on the solid phantom. The refractive index was assumed as 1.55 for the solid and 1.33 for the liquid.

One emission fiber and one detection fiber bundle were fixed at ρ = 3 cm. Their positions on the solid phantom were illustrated in [32,38]. When varying the depth, a perturbation was positioned in the midplane between the emitter and the detector, and it was moved in steps of 1 mm (2.5 mm) in the solid (liquid). When varying the lateral position, a perturbation was 1.5 cm (1.2 cm) below the surface and it was moved in steps of 2 mm (2 mm) in the solid (liquid). The position of a perturbation was defined as the center of a perturbation, as in [31,32]. A measurement was acquired at each position of a perturbation for 60 s (18 s) on the solid (liquid).

The contrasts that result from introducing a perturbation were calculated for five measurands: *N*, *m*_1_, *V*, and the number of photons in early and late time windows (from 0.5 to 1 ns for *N*_Eearly_ and from 3 to 4 ns for *N*_Late_). The zero time (0 ps) was defined at the maximum of the IRF. Measurands were calculated from DTOFs after background subtraction and using time bins that have photon counts ≥ 0.1% (2%) of the maximum for measurements on the solid (liquid) phantom. The contrasts were calculated as:

(1) $$\Delta A_{TW}(x)=-\ln(N_{TW}(x)/N_{TW,0}),$$

(2) $$\Delta m_{1}(x)=m_{1,0}-m_{1}(x),$$

(3) $$\Delta V(x)=V_{0}-V(x),$$

where $\Delta A_{TW}(x)$ is the change in the attenuation for a time window *TW* (*N*, *N*_Early_, and *N*_Late_) due to a perturbation located at *x*, which is either the depth or the lateral position. The zero subscript represents the reference measure when a perturbation was furthest away from the emitter and the detector.

Monte Carlo (MC) simulations were performed to obtain the sensitivity factors for the first three statistical moments: the mean partial pathlength (*MPP*), the mean time of flight sensitivity factor (*MTSF*), and the variance sensitivity factor (*VSF*). The MC code was developed by Wojtkiewicz *et al*. [44]. The sensitivity factors relate changes in moments to localized changes in *µ*_a_(*MPP* = Δ*A* / Δ*µ*_a_*, MTSF* = Δ*m*_1_ / Δ*µ*_a_, and *VSF* = Δ*V* / Δ*µ*_a_) at different depths and lateral positions, and hence they can be used for a comparison with the measured contrasts. For a detailed description of the sensitivity factors and their use, we refer to [44,45]. The MC parameters were chosen as in [46]. The model parameters were set to mimic the solid and the liquid inhomogeneous phantoms, using their optical properties obtained at 805 nm (values listed in Table 2).

### 3.4 Blood-lipid phantoms

Blood-lipid phantoms allow the assessment of a system’s capability to retrieve absolute concentrations of HbO_2_ and Hb, and hence total hemoglobin (HbT = HbO_2_ + Hb) and oxygen saturation (StO_2_ = HbO_2_ / HbT), during controlled dynamic StO_2_ changes.

The presented system was transported to University Hospital Zurich, University of Zurich, in Switzerland, to perform measurements on their blood-lipid phantom in parallel with a commercially available oximeter (OxiplexTSTM, frequency-domain, 692 and 834 nm, ISS, Champaign, Illinois, USA). OxiplexTS provides the absolute values of *µ*_a_ and *µ′*_s_ at two wavelengths, the concentrations of HbO_2_, Hb, and HbT, and StO_2_. A rigid sensor with four source-detector separations (2.5, 3.0, 3.5, and 4.0 cm) was used and the sampling rate was set to 1 s. Co-oximetry was used as another reference measure of HbT, by analyzing blood samples from the erythrocyte bag using a blood gas analyzer (ABL90 FLEX, Radiometer Medical ApS, Brønshøj, Denmark) and calculating the degree of dilution in the phantom, which is the same method as used in [47].

A detailed description of the phantom can be found in [34,35] and the following is a brief overview. The phantom and a similar protocol were used in previous studies that also used OxiplexTS [48–50]. The phantom consisted of a black 3D printed cubic container with windows on four sides, which allows simultaneously measuring with up to four systems. The windows were made from silicone and had tailored properties to mimic a layer of fat: *µ*_a_ = 0.063 cm^-1^, *µ′*_s_ = 6 cm^-1^ at 690 nm, and 1 mm thickness. The three main components that made up the liquid were: 2.5 L of phosphate-buffered saline (PBS, after Kreis, pH = 7.4, Kantonsapotheke Zurich, Zurich, Switzerland), a varying amount of Intralipid (Fresenius Kabi AG, Bad Homburg, Germany), and a varying amount of human blood from a human erythrocyte concentrate bag. Fresh baker’s yeast (3.0 g) was added to deoxygenate the phantom (StO_2_ = 0%) and the rate was increased by adding glucose 50% (AlleMan Pharma GmbH, Reutlingen, Germany). Blood was fully oxygenated (StO_2_ = 100%) by bubbling oxygen gas from an oxygen tank (typically for 1 min). Sodium bicarbonate buffer was added to keep the *pH* level close to 7.4. The temperature (37°C) and the mixing speed (500 rpm) were controlled with a hotplate that has a magnetic stirrer.

Measurements were repeated on two blood-lipid phantoms in which either blood or Intralipid was added at the end of each deoxygenation cycle before bubbling oxygen, i.e. when StO_2_ = 0%, similar as in [50]. The first phantom contained 74 ml of Intralipid and four amounts of blood: 20, 35, 55, and 70 ml. The second phantom contained 45 ml of blood and five amounts of Intralipid: 25, 50, 75, 100, and 125 ml. The refractive index was assumed as 1.33. The optical properties resulting from 74 ml of Intralipid and 45 ml of blood mixed with 2.5 L of saline are close to the typical optical properties of a neonatal brain (*μ′*_s_ ≈ 5.5 cm^-1^) [34].

### 3.5 In-vivo measurements

*In-vivo* measurements during cuff occlusions were performed to provide an example of a system’s ability to monitor hemodynamic responses in tissue. It is an easy-to-repeat test that allows checking the performance of oximeters *in-vivo* [51]. Measurements were repeated on three healthy volunteers: skinfold thickness (2.1, 2.4, and 2.5 mm), age (25, 26, and 29 years), one male. The measured blood pressure before the start of the experiment was around 120/80 mmHg. Written informed consent was filled in by participants.

A subject was relaxed in a Fowler’s position on a medical bed. A probe holder was attached on the posterior side of the left forearm, over the extensor muscles about midpoint between elbow and wrist. An arm-intended pneumatic cuff was loosely placed around the upper arm and rapid inflation was achieved with an electro-pneumatic regulator (SMC ITV2010-31F2N3). The inflation time was a few seconds; the recommended upper limit for a calf muscle was is 6 s [52]. 250 mmHg pressure was applied to occlude arterial and venous flow [25,38,53,54], and 60 mmHg pressure was applied to partially restrict venous flow without obstructing arterial flow [52,55]. The pressure was applied for 2 min. The refractive index of tissue was assumed as 1.4 [56].

### 3.6 TD-NIRS data analysis

Most measurements were performed with two detection modules at two source-detector distances (ρ = 2 and 3 cm, or 3 and 4 cm) away from the same emission fiber. The acquisition time was 0.3 s and the sampling rate was 3 Hz. During post-processing, 3 consecutive DTOFs were summed to increase the signal-to-noise ratio, resulting in a sampling rate of 1 s.

*µ_a_* and *µ′*_s_ were estimated using the curve fitting method [57,58] as implemented in NIRFAST software [59]. It is considered a gold standard method to determine the absolute optical properties. A homogeneous semi-infinite medium model under extrapolated boundary conditions was used. The fitting region of the DTOFs was limited based on the percentage of its maximum, i.e. from 85% on the rising edge to 1% on the tail for phantom measurements (Section 4.2), and from 75% to 3% for blood-lipid phantoms and *in-vivo* measurements (Sections 4.4 and 4.5).

The concentrations of HbO_2_ and Hb were calculated using the estimated *µ*_a_ at multiple wavelengths and the Beer-Lambert law. For *in-vivo* measurements, another method was also used to calculate the changes in concentrations relative to a baseline, and the results were compared to the first method, as in [60]. The second method uses changes in light attenuation and the modified Beer-Lambert law (MBLL). The mean optical pathlength was calculated for each spectral channel using the method demonstrated by Delpy *et al*. [61], which relies on the measured mean time of flight and the speed of light in tissue. Zhao *et al*. [62] compared the hemoglobin absorption spectra from different sources and found that the most consistent performance was achieved with the values provided by Moaveni *et al*. [63], which were used in this study. We used the absorption spectra of water provided by Matcher *et al*. [64]. The concentration of water was assumed constant and the contribution of water’s *µ*_a_ for the estimation of absolute concentrations was addressed as follows. The concentrations of three chromophores (HbO_2_, Hb, and water) were estimated in the first step. A mean value of water concentration over the whole measurement was calculated and its contribution to the measured *µ*_a_ was subtracted. Finally, the concentrations of only two chromophores (HbO_2_ and Hb) were estimated using the water-subtracted *µ*_a_. Resolving for concentrations of only two chromophores leads to less noisy results. The concentrations were calculated using 11 spectral channels that covered the spectral range from 705 to 830 nm.

## 4. Results and discussions

Results were similar for both detection modules and hence some results are shown for only one.

### 4.1 BIP protocol

**Fig. 2.**
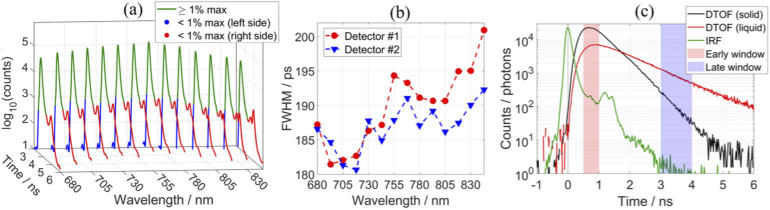
(a) The IRFs for 14 spectral channels after background subtraction, and (b) the FWHM of the IRFs. (c) The IRF and the DTOFs measured on solid and liquid inhomogeneous phantoms that were used for the nEUROPt protocol, after background subtraction, at 805 nm. Each IRF and DTOF contains about 10^6^ photons.

Figure 2 (a) shows the IRFs for 14 spectral channels measured with one detection module and Fig. 2 (b) reports the corresponding FWHM for both detection modules. Figure 2 (c) shows the IRF at 805 nm and the DTOFs measured on two inhomogeneous phantoms that were used for the nEUROPt protocol (before introducing a perturbation). Similar DTOFs for the same solid phantom were reported in [25]. The IRFs match the reported IRF for the same PML detector [39] since the FWHM is close to 150 ps and there is a small afterpulse peak (1 to 2% of the maximum) about 1 ns after the main peak. It is common for conventional PMTs to have such afterpulse peaks, which are probably due to photoelectrons that are reflected at the first dynode but then forced back to the dynode by the electric field, page 169 in [39]. The reported FWHM of multi-wavelength TD-NIRS systems are typically broader, e.g. 465 ps [19], 370 [38], 230 ps [53], and 83 to 263 ps depending on the detector [15], at around 800 nm. The narrowest reported IRF (83 ps) was obtained with a detector that has a much smaller active area (0.1 mm diameter) and hence a much lower responsivity.

The ε_DNL_ was found to be 3% in the interval between 1 and 10 ns; the maximum deviation of the photon count was 1.5%. This is consistent with the previously reported measures of ε_DNL_, e.g. 6.77% [16] and 3.5% [24]. The warmup period was found to be between 30 to 60 min, during which time the parameters of the IRF can change drastically. Afterward, long-term drifts can be observed during the next few hours (up to about 5 hours), which can be accounted for by periodically measuring the IRF (e.g. every 30 min). The duration of most clinical measurements is typically less than 1 hour, in which case, after a 30 to 60 min warmup period, it is sufficient to measure the IRF at the start and at the end, i.e. before and after attaching probes to a patient. The two IRFs can be used in data analysis to remove drift in *N*, *m*_1_, and *V*, by assuming that the drift was constant over time. Also, the first and second IRFs can be used for analyzing the first and second half of the measurement, respectively. A more rigorous approach to account for drifts involves directing a small portion of the laser beam to the detector along with the signal from the tissue [15], which can be adapted in the next version of the system.

The calculated temporal fluctuations of moments, which were measured on phantom B2, were about: ±0.2% for *N*, ±1.0 ps for *m_1_*, and ±700 ps^2^ for *V*, at 805 nm. The values at other wavelengths were similar. These reported standard deviations contain the photon noise, which depends on the count rate and shape of the DTOF, and the instrumental noise, which depends on the system [65].

### 4.2 MEDPHOT protocol

**Fig. 3.**
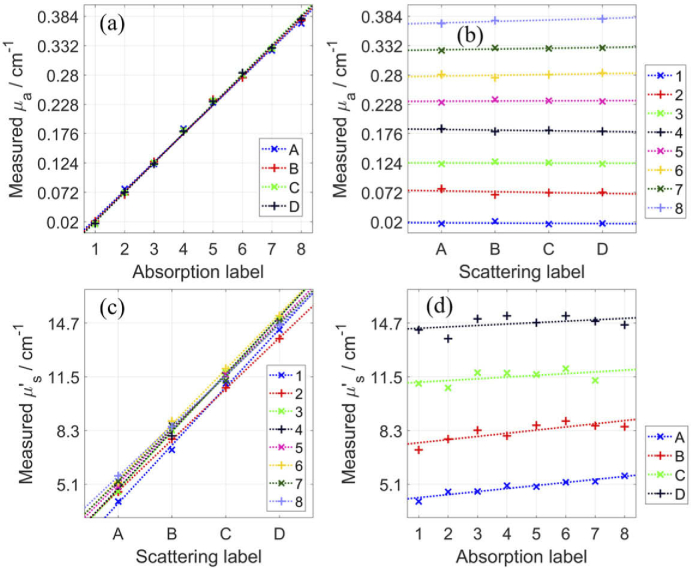
The obtained *µ*_a_ and *µ′*_s_ of the 32 phantoms, which make up all combinations of four nominal *µ′*_s_ values (from 5 to 20 cm^-1^ in steps of 5 cm^-1^, labels from A to D) and eight nominal *µ*_a_ values (from 0 to 0.35 cm^-1^ in steps of 0.05 cm^-1^, labels from 1 to 8). These scatter plots can be used to assess the deviation from linearity for *µ*_a_ (a) and *µ′*_s_ (c), and the coupling of Δ*µ′*_s_ to Δ*µ*_a_ (b) and Δ*µ*_a_ to Δ*µ′*_s_ (d). The shown values are for ρ = 2 cm and 805 nm.

Figure 3 shows the four scatter plots that illustrate the linearity and the coupling of *µ*_a_ and *µ′*_s_ (for ρ = 2 cm and 805 nm). Qualitatively, the scatter plots illustrate an exemplary level of linearity and coupling, for both *µ*_a_ and *µ′*_s_. The same was observed for ρ = 3 cm and for other wavelengths (not shown). Important to note, for measurements at ρ = 3 cm, the signals were too low for some of the more absorbing phantoms and hence phantoms with nominal *µ*_a_ ≥ 0.25 cm^-1^ (labels 6 to 8) were excluded. For measurements at ρ = 2 cm, 31 phantoms were used, excluding only phantom C8. The power of the laser was within the safe limit for measurements on patients (2 mW/mm^2^), although it could be increased for phantom measurements.

The mean deviations from linearity and the coupling are reported in Table 1 for two ρ (2 and 3 cm) and two wavelengths (705 and 805 nm). The results are presented for each subset of the 32 phantoms, which provides more information about the system’s performance and also allows comparing with other systems that measured on only a subset of phantoms. Measurements on phantoms with the lowest nominal *µ′_s_* (5 cm^-1^, label A) and lowest nominal *µ*_a_ (0 cm^-1^, label #1) have the highest deviations from linearity for ρ = 2 cm but not so much for ρ = 3 cm. When estimating low values of optical properties, even small deviations have a high magnitude in units of percentage. Increasing ρ reduces the uncertainty due to the finite width of time bins [40]. Furthermore, the deviation from linearity will be lower if using fewer phantoms since a straight line is easier to fit with fewer data points, while for the same reason the coupling of Δ*µ*_a_ to Δ*µ′*_s_ will be worse if using fewer phantoms. This is observed when comparing the results for ρ = 2 cm (31 phantoms were used) and ρ = 3 cm (20 phantoms were used). The coupling is similar for different phantom sets, except for those that had a low signal level (at ρ = 3 cm on phantoms with nominal *µ*_a_ = 0.20 cm^-1^, label 5). The coupling could be in part due to the theoretical model that was used for the fitting method.

The overall measure of linearity and coupling can be taken as the mean of different phantom sets. For ρ = 2 cm and 805 nm, the mean deviation from linearity is 4.3% for *µ*_a_ and 1.4% for *µ′*_s_, and the mean coupling is 2.8 for Δ*µ_a_* to Δ*µ′*_s_ and 2.2 × 10^−4^ for Δ*µ′*_s_ to Δ*µ*_a_. In other words, if *µ′*_s_ increases by ∼1 cm^-1^, the system and the used method will show an increase also in *µ_a_* by ∼2.2 × 10^−4^ cm^-1^. The obtained linearity is consistent with previously reported systems, e.g. mean deviation of 4.2% for *µ*_a_ and 4.9% for *µ′*_s_ [29]. The linearity and coupling reported in [33] were calculated using the median instead of the mean of different phantom sets, which lead to smaller values of linearity (as low as 1% for *µ*_a_ and 1.5% for *µ′*_s_) although similar values of coupling (between 1 and 10 for Δ*µ*_a_ to Δ*µ′*_s_ and between 2 × 10^−4^ and 15 × 10^−4^ for Δ*µ′*_s_ to Δ*µ*_a_ for the 29 instruments that were enrolled in the BitMap exercise). The obtained coupling is consistent with the best performance of the reported TD-NIRS systems.

**Table 1.** The mean deviation from linearity (%) and the coupling (unitless), for *µ*_a_ and *µ′*_s_ obtained on the 32 phantoms, at two ρ and two wavelengths. Phantoms with nominal *µ*_a_ ≥ 0.25 cm^-1^ (labels 6 to 8) were excluded for ρ = 3 cm due to low signals.

	ρ = 2 cm		ρ = 3 cm		ρ = 2 cm		ρ = 3 cm	
	705 nm	805 nm	705 nm	805 nm	705 nm	805 nm	705 nm	805 nm
	**Mean deviation in *µ_a_* (%) from linearity**				**Coupling of *Δµ_a_* to Δ*µ′_s_***			
A	7.8	6.5	3.6	1.8	3.1	3.6	6.8	7.7
B	3.2	3.1	2.9	1.0	4.1	3.8	4.4	5.3
C	2.5	4.1	5.5	1.1	0.9	2.1	3.0	2.4
D	3.6	3.5	3.8	2.5	0.1	1.7	1.7	3.1
**Mean**	**4.3**	**4.3**	**4.0**	**1.6**	**2.0**	**2.8**	**4.0**	**4.6**
	**Mean deviation in *µ′_s_* (%) from linearity**				**Coupling of Δ*µ′_s_* to Δ*µ_a_* (×10^−4^)**			
1	4.8	2.2	4.7	4.3	0.2	0.9	1.1	1.1
2	0.2	0.6	2.5	2.4	2.4	2.9	2.0	0.1
3	1.5	1.3	2.4	1.6	4.4	0.3	3.3	6.8
4	1.7	1.9	2.2	3.7	1.8	2.4	0.3	4.8
5	2.8	1.9	2.8	1.9	1.5	0.7	5.6	12.0
6	2.6	1.5	-	-	1.0	2.9	-	-
7	1.0	1.4	-	-	5.3	2.7	-	-
8	0.7	0.4	-	-	5.7	5.2	-	-
**Mean**	**1.9**	**1.4**	**2.9**	**2.8**	**2.8**	**2.2**	**2.5**	**5.0**

The calculation of linearity requires an array of phantoms with linear increments of *µ*_a_ and *µ′*_s_. The calculation of coupling requires also a rough estimate of the step size of the increments. Therefore, similar results could be obtained using e.g. a liquid phantom as in [66], where *µ*_a_ and *µ′*_s_ were linearly increased by adding ink and Intralipid, respectively. The assessment of linearity and coupling can be enhanced if the true optical properties of the 32 phantoms get established.

**Fig. 4.**
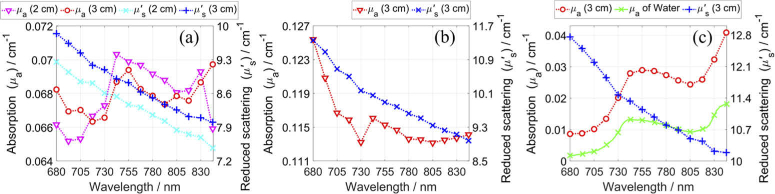
Obtained spectra of *µ*_a_ and *µ′*_s_ for phantom B2 (a), solid inhomogeneous phantom (b), and 1% aqueous solution of Intralipid (c). The spacing between data points is 12.5 nm and the dashed lines help guide the eye. The *µ*_a_ of water was taken from [64] and plotted with 1 nm spacing.

Here, we report the system’s accuracy of retrieving the absolute *µ*_a_ and *µ′*_s_. The values shown in Fig. 3 match the values in similar scatter plots in [23,25,27,67] for a similar wavelength. The obtained values for ρ = 3 cm are similar and one example is shown in Fig. 4 (a). However, due to a lack of true values for the 32 phantoms, we focus on the three well-characterized phantoms that are commonly used for assessing a system’s performance. Figure 4 shows the spectra of the obtained *µ*_a_ and *µ′*_s_ for the three phantoms, and Table 2 reports these values for two ρ along with the previously reported values around the same four wavelengths. Note, most referenced values were roughly estimated from the reported spectral figures for the chosen four wavelengths. The results for phantom B2 match with the previously reported spectra [27–29] since *µ′*_s_ is close to 10 cm^-1^ and decreases with wavelength and *µ*_a_ is close to 0.07 cm^-1^ at all wavelengths. The obtained values of *µ*_a_, and especially *µ′*_s_, are slightly lower than the previously reported, but consistent with reproducibility of modern TD-NIRS systems, e.g. maximum day-to-day variations with respect to the mean: -5% to +6% for *µ*_a_ and -4% to +3% for *µ′*_s_ [25]. Similarly, the results for the solid inhomogeneous phantom match the spectra reported in [32], although now the obtained values of *µ′*_s_, and especially *µ*_a_, are slightly higher than the previously reported. Figure 4 (c) shows the results for a 1% aqueous solution of Intralipid alongside the reported *µ*_a_ of water. The shape of the obtained *μ*_a_ spectra matches that of water, which supports that the system’s spectral channels correspond to the specified wavelengths. The *µ*_a_ and *µ′*_s_ values are close to those reported in [15,19,43] for four wavelengths, and to those reported in [68] for 760 nm (*µ*_a_ = 0.029 cm^-1^ and *µ′*_s_ = 10 cm^-1^). The system and the used method can retrieve spectra of *µ*_a_ and *µ′*_s_ with high accuracy, as compared to the previously reported spectra.

**Table 2.** The values of *μ*_a_ and *μ′*_s_ at four wavelengths for the three phantoms that are shown in Fig. 4, alongside the results that were previously shown for other systems.^*a*^

*μ*_a_ / cm^-1^				*μ′*_s_ / cm^-1^				ρ / cm	Ref.
∼705	∼755	∼805	∼830	∼705	∼755	∼805	∼830		
*Phantom B2*									
0.065	0.070	0.068	0.069	8.8	8.4	7.8	7.7	2	Fig. 4 (a)
0.067	0.069	0.068	0.069	9.4	8.8	8.3	8.1	3	Fig. 4 (a)
∼0.07^690^	∼0.07^730^	∼0.07^800^	∼0.07^830^	∼10.5^690^	∼10^730^	∼9^800^	∼8.5^830^	∼2.4	[27]
∼0.07	∼0.07	∼0.07	∼0.07	∼11	∼10	∼9	∼8	2	[29]
0.078^685^	0.076^760^	-	0.073^820^	11.5^685^	9.7^760^	-	8.7^820^	1.5	[25]
∼0.075	∼0.075	∼0.075	-	∼12	∼11	∼10	-	2	[28]
*Solid dynamic phantom*									
0.117	0.115	0.113	0.114	10.7	9.9	9.3	9.1	3	Fig. 4 (b)
∼0.10	∼0.10	∼0.09	∼0.09	∼9.5	∼8.5	∼8	∼7.5	3	[32]
*1% aqueous solution of Intralipid*									
0.010	0.029	0.024	0.033	12.2	11.1	10.5	10.2	3	Fig. 4 (c)
∼0.01	∼0.03	0.026^800^	∼0.04	∼12.5	∼11.5	∼10.5	10.38^830^	2	[19]
-	∼0.03	∼0.025	∼0.035	n.a.	n.a.	n.a.	n.a.	1 to 3	[43]

^*a*^ n.a.: Referenced work used other concentrations of Intralipid (0.94% to 4.00%), which resulted in different *µ′_s_*.

### 4.3 nEUROPt protocol

**Fig. 5.**
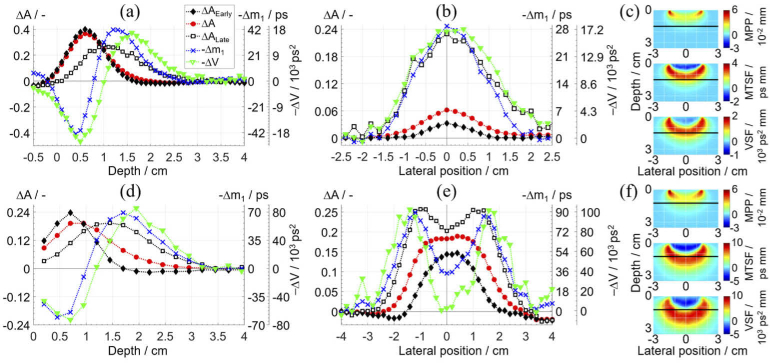
Contrasts of five measurands (ΔA_Early_, ΔA, ΔA_Late_, Δm_1_, and ΔV) during depth scans (a, d) and lateral scans (b, e) measured on the solid (top row) and the liquid (bottom row) inhomogeneous phantoms. The lines are drawn to guide the eye. The sensitivity factors (MPP, MTSF, and VSF) (c, f) illustrate the expected behavior for ΔA, Δm_1_, and ΔV; the horizontal black lines show the depth of a perturbation during lateral scans. The presented values were obtained at 805 nm.

Figure 5 shows the obtained contrasts of five measurands and the sensitivity factors (SF) of three statistical moments, for the two inhomogeneous phantoms. The quantitative results are summarized in Table 3. The findings for the two phantoms are similar, except for the liquid phantom the contrasts reach deeper and have a higher magnitude.

**Table 3.** Quantitative parameters of the contrasts in Fig. 5.

	Maximum contrast		Contrast at 1.5 cm (1.2 cm for liquid)		FWHM / cm
Measurand	Depth / cm	Magnitude	Depth scan	Lateral scan	
*Solid inhomogeneous phantom, data in Fig. 5 (a, b)*					
Δ*A_Early_*	0.6	0.400	0.025	0.033	1.1
Δ*A*	0.6	0.366	0.057	0.062	1.5
Δ*A_Late_*	1.0	0.278	0.196	0.230	2.2
Δ*m_1_* / ps	1.2	41.9	37.3	28.7	1.9
Δ*V* /10^3^ ps^2^	1.6, ∼1.6 [31,42]	16.9, ∼20 [31,42]	15.3	16.9	2.3
*1% aqueous solution of Intralipid, data in Fig. 5 (d, e)*					
Δ*A_Early_*	0.70	0.237	0.112	0.144	2.2
Δ*A*	0.70 to 0.95	0.192	0.164	0.183	3.1
Δ*A_Late_*	1.45	0.194	0.188	0.203	-
Δ*m_1_* / ps	1.70	68.9	35.1	34.2	-
Δ*V* / 10^3^ ps^2^	1.95	85.6	2.2	2.5	-

The contrasts for moments agree with SF and with their known behavior [45] since the position of the maximum contrast is deeper for higher order moments and the FWHM of the lateral scan increases following the broadening of ‘banana’ shapes. The moments *m*_1_ and *V* increase when a perturbation is positioned near the surface and decrease when it is positioned deeper; this effect has been well described [45]. Oddly, the contrasts for Δ*A* can be negative at the smallest depths (the same was reported in [23,24,53]), which corresponds to an increase in the photon count rate after introducing a perturbation, although at these depths a perturbation is outside of the phantom (negative depth). The phantom inherently has a weak heterogeneity in the structure due to a movable part, and importantly, it was found that a homogeneous rod causes a noticeable perturbation [32]. Also, the measurement geometry is not semi-infinite because the rod extends beyond the phantom’s surface. These reasons could have contributed to the difference between the obtained Δ*m*_1_ (37.3 and 28.7 ps, shown in Table 3) during two scans when a perturbation was at the same depth and lateral position. These reasons are not present for the liquid phantom and the obtained Δ*m*_1_ was similar during two scans (35.1 and 34.2 ps). The measure of Δ*A* is more prone to a slight drift, as seen in Fig. 5 (e) by comparing the values at -4 and 4 cm. Also, the sensitivity of Δ*A* is focused at a shallow depth, which is a known challenge for continuous-wave NIRS [69]. The results for *ΔV* are consistent with previous studies on similar phantoms [31,42].

The contrasts of the early Δ*A*_Early_ and late Δ*A*_Late_ time windows confirm that the later photons on average travel deeper and hence have a higher sensitivity to deeper perturbations. For an ideal system, the latest time windows could probe well beyond a depth of 3 cm [70]. However, an IRF with a substantial late afterpeak can have a devastating effect for time windows [31], although the changes in moments are independent of the IRF if the time range encompasses the whole DTOF [45]. The presented system’s IRF allows to confidently use time windows and relevant methods, as confirmed by the IRF parameters (Section 4.1) and Δ*A*_Late_ in Fig. 5.

### 4.4 Blood-lipid phantoms

**Fig. 6.**
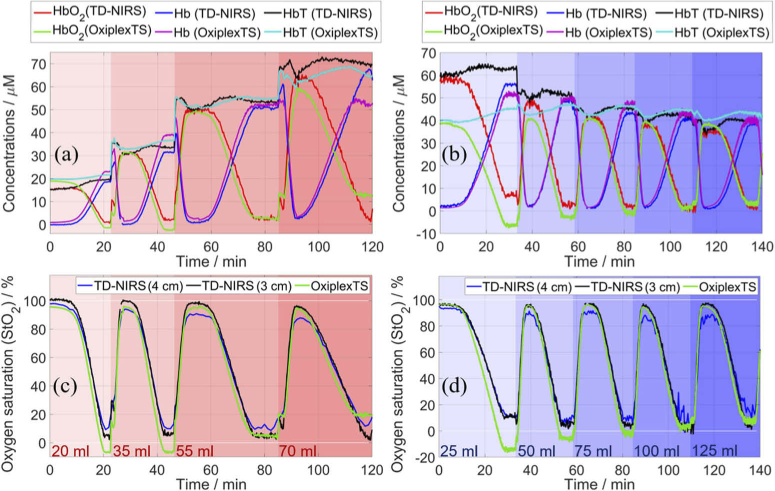
Measurements on two blood-lipid phantoms that contained a varying amount of blood (left panels) or Intralipid (right panels); the amount is expressed as the background color and written in the bottom panels. Concentrations of HbO_2_, Hb, and HbT obtained with TD-NIRS at 3 cm and OxiplexTS (a, b). StO_2_ obtained with TD-NIRS at 3 and 4 cm and OxiplexTS (c, d). For displaying purposes, 10 data points were averaged for TD-NIRS at 3 cm and OxiplexTS, and 30 data points were averaged for TD-NIRS at 4 cm due to higher noise.

Figure 6 shows time traces of the obtained concentrations and StO_2_ for two blood-lipid phantoms during dynamic StO_2_ changes. The concentrations obtained with the presented system (TD-NIRS) at 3 and 4 cm are similar, and the values for ρ = 3 cm are shown because they contain lower noise. The signal level, i.e. photon count rate, greatly reduced after adding blood, which is a consequence of an increase in *µ_a_*.

The bubbling of oxygen can form bubbles inside the phantom, which then dissipate over time. In Fig. 6 (a, b), HbT always drops by a few µM during the bubbling of oxygen and afterward gradually returns to the initial value (prior to bubbling). The magnitude of the drop is higher for a higher blood concentration and reaches up to ∼5 µM for TD-NIRS at 3 cm and OxiplexTS, and up to ∼10 µM for TD-NIRS at 4 cm. The time to return to the initial value is about 12 min for TD-NIRS and always longer for OxiplexTS. The chosen zero-time is after oxygen was bubbled, and hence the first cycles show an increasing HbT as it returns to the value that it had. Apart from this dip, HbT remains constant during all desaturation cycles as intended.

The concentration of oxyhemoglobin HbO_2_ obtained with TD-NIRS at 3 cm approaches 0 µM at the end of all desaturation events, except at the lowest scattering (25 ml Intralipid) where it is always above 5 µM. Consequently, the corresponding StO_2_ is in the range from 100% to 0%, except at the lowest scattering where it is always above 10%. The amount of blood and Intralipid have no apparent effect on the estimation of StO_2_ with TD-NIRS at 3 cm, except for the lowest amount of Intralipid. TD-NIRS at 3 and 4 cm obtain similar values of Hb, but the values of HbO_2_ for 4 cm are slightly lower at the maximum and higher at the minimum compared with the values for 3 cm, which is amplified for the following cycles. Consequently, StO_2_ for 4 cm is in the range from 95% to 10% during the first cycle, and the maximum decreases reducing the range with each addition of blood or Intralipid. Also, StO_2_ for 4 cm is significantly noisier, which is visible in Fig. 6 despite the higher temporal averaging. Under most conditions, the system reliably retrieves concentrations and StO_2_ with good precision.

For an assessment of the accuracy, we compare TD-NIRS with the results of OxiplexTS and co-oximetry. The closest match between the TD-NIRS and the OxiplexTS results is observed for 55 ml of blood (Fig. 6 (a, c)) and 75, 100, and 125 ml of Intralipid (Fig. 6 (b, d)). The largest difference is observed for the lowest concentrations of blood (20 and 35 ml) and Intralipid (25 and 50 ml), where OxiplexTS obtained values slightly below zero. When blood or Intralipid is increased, for the results of OxiplexTS, the maximum of HbO_2_ remains the same while the minimum increases, such that HbO_2_ and hence StO_2_ become non-negative and match closer with TD-NIRS at 3 cm. The previously reported measurements with OxiplexTS on a similar phantom do not have negative values [50], which is probably because the authors used windows that mimick a neonatal head (4 mm thickness, *µ_a_* = 0.10 cm^-1^, and *µ′*_s_ = 9.6 cm^-1^) and the windows in the present study mimick a layer of fat (1 mm thickness, *µ_a_* = 0.063 cm^-1^, and *µ′*_s_ = 6 cm^-1^).

Table 4 reports the mean values of HbT during the plateau regions at the end of desaturation events, which lasted a few minutes, and the results of co-oximetry. The standard deviations of these values are negligible, except for TD-NIRS at 4 cm (∼1 µM). OxiplexTS values are close to the results of co-oximetry; the biggest discrepancies are for the highest concentrations of blood and Intralipid. The values of TD-NIRS at 4 cm match with OxiplexTS and co-oximetry within a few percent, except at the lowest concentration of Intralipid where TD-NIRS overestimated HbT. The values of TD-NIRS at 3 cm are only slightly lower than the values at 4 cm. In summary, the system can retrieve HbO_2_, Hb, HbT, and StO_2_ with an accuracy that is consistent with OxiplexTS and co-oximetry, except for the lowest concentration of Intralipid.

**Table 4.** Comparison of HbT / (µM) obtained using different instruments.

Blood	20 ml	35 ml	55 ml	70 ml	———————- 45 ml ————————-				
Intralipid	————— 74 ml —————				25 ml	50 ml	75 ml	100 ml	125 ml
Co-oximetry	21.3	37.0	57.3	72.1	48.1	48.6	48.6	48.4	48.3
OxiplexTS	21.6	36.6	54.7	65.3	45.1	46.8	46.3	44.5	43.1
TD-NIRS (4 cm)	21.4	36.7	56.6	73.0	62.0	50.7	47.7	42.5	42.9
TD-NIRS (3 cm)	19.6	33.5	53.4	69.9	62.9	50.6	45.1	41.9	39.6

**Fig. 7.**
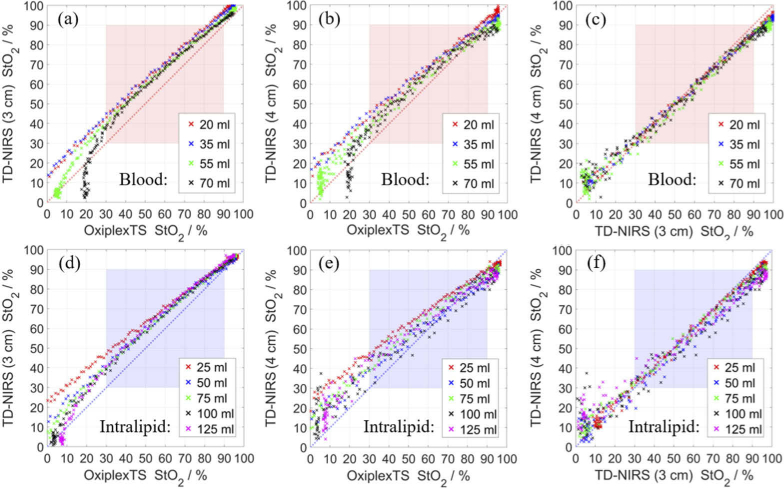
Comparison of three traces of StO_2_ in Fig. 6. TD-NIRS at 3 cm versus OxiplexTS (Left panels). TD-NIRS at 4 cm versus OxiplexTS (Middle panels). TD-NIRS at 4 cm versus at 3 cm (Right panels). The legends correspond to the amount of blood or Intralipid inside the phantom. Linear fits were obtained for data points 30% < StO_2_ < 95% and reported in Table 5.

The StO_2_ data in Fig. 6 were resampled to 10 s on a common time base for comparing the values obtained by different systems during deoxygenation events. The StO_2_ values obtained with TD-NIRS are almost always higher than obtained with OxiplexTS (Fig. 7(a, b, d, e)), which could be observed also in Fig. 6. The values obtained with the two systems better match at higher StO_2_, which was also found for most systems that were similarly compared with OxiplexTS in [34,35]. Increasing the amount of blood brings the data points closer to the unity line, and varying the amount of Intralipid (excluding 25 ml) does not significantly affect the results. For the TD-NIRS measurements at 3 cm versus 4 cm (Fig. 7(c, f)), most data points are slightly above the unity line at low StO_2_ and below the unity line at high StO_2_.

**Table 5.** Coefficients for linear transformation of data in Fig. 7: $StO_{2}(Device\#1)=a\times StO_{2}(Device\#2)+b$ . The parameters include intercept (*b*), slope (*a*), and coefficient of determination (*R^2^*).

Device #1 →Device #2 →		TD-NIRS (3 cm)OxiplexTS			TD-NIRS (4 cm)OxiplexTS			TD-NIRS (4 cm)TH-NIRS (3 cm)		
Blood	Intralipid	*b*	*a*	*R^2^*	*b*	*a*	*R^2^*	*b*	*a*	*R^2^*
20 ml	74 ml	17.1	0.89	0.9980	19.7	0.82	0.9945	4.1	0.91	0.9936
35 ml	74 ml	19.2	0.86	0.9980	22.4	0.77	0.9953	5.3	0.89	0.9936
55 ml	74 ml	14.5	0.89	0.9972	14.1	0.84	0.9841	0.3	0.95	0.9858
70 ml	74 ml	12.8	0.91	0.9947	14.2	0.83	0.9794	2.5	0.92	0.9837
45 ml	25 ml	25.5	0.75	0.9973	26.9	0.71	0.9934	2.8	0.95	0.9910
45 ml	50 ml	16.9	0.84	0.9923	17.1	0.76	0.9680	2.1	0.89	0.9670
45 ml	75 ml	14.6	0.87	0.9980	18.1	0.79	0.9821	4.8	0.90	0.9845
45 ml	100 ml	14.2	0.88	0.9947	15.6	0.78	0.9360	3.8	0.87	0.9198
45 ml	125 ml	16.1	0.87	0.9960	21.3	0.73	0.9769	8.1	0.83	0.9745

The differences between the traces of the obtained StO_2_ can be quantified using the approach proposed by Kleiser *et al*. [34,35]: apply a first degree polynomial fit (lowest least square error) to the data in Fig. 7 for values of StO_2_ from 30 to 90% (Kleiser *et al.* used 15 to 95%). The obtained parameters of the fit are presented in Table 5 for each deoxygenation cycle. There is no noticeable dependence on the concentration of blood and Intralipid. The slope is always lower than 1 and the y-intercept is always positive. The fits are better than for most systems that were used in [34,35].

The duration of deoxygenation cycles ranged from 23 to 38 min (mean 28 min) and it depended mostly on the added amounts of glucose and blood. A previous study showed a significant decrease in *μ′*_s_ (around 2 cm^-1^) following deoxygenations inside a similar phantom [48]. The *μ′*_s_ (obtained with TD-NIRS and OxiplexTS, not shown) was stable in both phantoms and decreased by an insignificant amount. The greatest reduction (about 0.2 cm^-1^ in 30 min) was during the last deoxygenation cycle that had the highest amount of blood. Similar results for Fig. 6 and 7 and Tables 4 and 5 were obtained using a subset of spectral channels of the TD-NIRS system, as long as the first and last channels were included, apart from an increased level of noise when using fewer channels.

### 4.5 In-vivo measurements

**Fig. 8.**
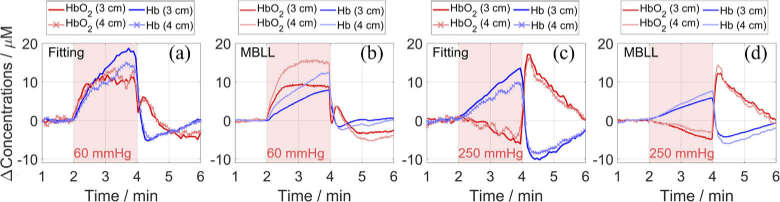
Changes in the concentrations of HbO_2_ and Hb obtained *in-vivo* at two ρ (3 and 4 cm) on the forearm of one subject, during a venous (a, b) and an arterial (c, d) cuff occlusion. The results were obtained with the fitting method (a, c) and the MBLL method (b, d). The red background represents the start and end of a 2-min occlusion period. The results of the fitting method were smoothed for displaying purposes: a third order median filter (13.8 s) for ρ = 4 cm, and a smoothing filter (1.5 s) for both ρ.

Figure 8 shows the retrieved dynamic changes in concentrations, which were calculated using the fitting method (time-domain information) and the MBLL method (intensity information), during arterial and venous cuff occlusions on the forearm of one subject. For the other two subjects, the following findings were also observed. During the venous occlusion (Fig. 8(a, b)), both HbO_2_ and Hb increase at two rates, first rapidly and then much slower (close to 0 for the shown subject, and even negative for HbO_2_ for another subject). The observed changes are consistent with the physiology of a venous occlusion [52,55]. During the arterial occlusion (Fig. 8(c, d)), the obtained increase in Hb is greater than the decrease in HbO_2_, which leads to an increase in HbT similar as found in [19,38,53,54,56]. After releasing the pressure, the concentrations ‘overshoot’ and then slowly return to baseline. The expected hemodynamic responses for both occlusions were retrieved using measurements at either ρ = 3 or 4 cm.

The measurements at two ρ lead to similar changes in concentrations if using the fitting method, but not if using the MBLL method. The magnitudes are higher for ρ = 4 cm and closer to the results of the fitting method. A difference in the trend is noticeable in Fig. 8 (b), where HbO_2_ for 3 cm decreases between 2.5 and 4 min but HbO_2_ for 4 cm remains constant. An arm is a heterogeneous medium and greater hemodynamic changes are expected to occur in the deeper tissues, i.e. in muscles. The sensitivity is more focused inside the superficial layer for the MBLL method compared to the fitting method [68] and depth sensitivity can be improved for both methods by increasing ρ.

## 5. Discussion and future perspectives

This work demonstrated the performance of the system, which is intended for muscle and brain monitoring in a clinical environment. The IRF, the temporal fluctuations, and the warmup time confirm the good specifications of modern electronics (BIP protocol). The warmup time must be considered when planning clinical measurements, and a good IRF is necessary for using time windows and relevant methods [31]. The linearity for *µ*_a_ and *µ′*_s_, the coupling between Δ*µ*_a_ and Δ*µ′*_s_, and the accuracy of retrieving *µ*_a_ and *µ′*_s_ were quantitatively assessed (MEDPHOT protocol). The results are consistent with the best performances of previously reported multi-wavelength TD-NIRS systems for a big range of *µ*_a_ and *µ′*_s_; the nominal optical properties of the tested phantoms ranged from 0 to 0.04 cm^-1^ for *µ*_a_ and from 5 to 20 cm^-1^ for *µ′*_s_. The contrasts measured on the solid inhomogeneous phantom, which has *µ*_a_ and *µ′*_s_ similar to tissue, confirm the system’s sensitivity at great depths for measurements on tissues (if tissue has similar *µ*_a_ and *µ′*_s_) since the obtained Δ*V* is non-zero at up to ∼2.5 cm depth. The contrasts measured on the liquid inhomogeneous phantom, which had a much lower *µ*_a_ but similar *µ′*_s_, showed a different behavior of measurands but which was consistent with the theory as illustrated with Monte Carlo simulations. Although using later time windows improves depth sensitivity, it also increases noise which hinders the overall performance of detecting deeper changes in *µ*_a_ [46].

Blood-lipid phantom measurements demonstrated the system’s ability to reliably and accurately retrieve absolute concentrations of hemoglobin, and hence StO_2_, for phantoms with a varying amount of blood and Intralipid. The results obtained using the reported system (TD-NIRS) are close to the reference measurements, which were obtained using OxiplexTS and co-oximetry. The biggest discrepancy is for the lowest amount of Intralipid, which corresponds to *µ′*_s_ lower than a typical biological tissue. Improvements in the method of retrieving optical properties could improve the results. The used blood-lipid phantom is well suited for assessing the performance of oximeters: the ingredients can be precisely varied, the controlled deoxygenation cycles are highly repeatable, and up to four systems can measure in parallel without experiencing cross-talk. The used windows were mimicking a layer of fat, but they could be replaced to mimic other layers, e.g. neonatal head [48]. Previous studies have reported that occasionally the scattering properties of blood-lipid phantoms change over time, which is addressed in [48] and where possible reasons are discussed. In the present work, *µ′*_s_ was obtained by both NIRS systems and it did not change significantly over time. The challenges of validating oximeters were described in detail in a review paper [71], including the pros and cons of *in-vitro* and *in-vivo* methods. Recently, Kovacsova *et al.* [72] presented a novel algorithm for measuring StO_2_ and compared its performance to other published algorithms using data from a blood-lipid phantom that was developed in their group, as well as *in-vivo* data on a neonate, which demonstrates an example of another blood-lipid phantom and the latest improvements in the assessment of StO_2_.

The description of the system explained how 32 DTOFs (16 spectral channels and 2 detection modules) get acquired at up to 3 Hz. A drawback of measuring at 16 wavelengths with a single TCSPC card is the limited count rate, e.g. because of the pile-up effect, which inherently limits the count rate per wavelength compared to measuring at a single wavelength. The optical power is spread over a chosen wavelength range and the current version of the system cannot redistribute it to specific, desired wavelengths. For accurately estimating concentrations of chromophores, the extinction coefficients can be modified to account for the finite spectral bandwidth [73]. An advantage of the system is the excessive number of spectral channels and the fast sampling rate, which combined, may potentially allow monitoring the oxidation state of the CCO enzyme. The time-domain information allows applying depth-resolved methods for separating signals that come from different depths, e.g. brain and extracerebral tissue. Additionally, different spectral channels have slightly different depth profiles, which was exploited by Gerega *et al*. [37] to estimate changes in concentrations of ICG in many layers. The depth-resolved assessment will be investigated in the future and it has the potential to overcome the problems related to the extracerebral layer [69], which will make the measurements more reliable as well as more informative of the cerebral tissue.

The system is well suited for detecting fluorescence during an injection of Indocyanine Green [37], which allows assessing brain perfusion. The current study supports using the system also for other applications that involve monitoring constant and changing optical properties of tissue, e.g. tissue oximetry, tissue spectroscopy, and functional imaging.

## 6. Conclusions

We presented a description of the multi-wavelength time-domain NIRS system that obtains 32 DTOFs at up to 3 Hz. The quantitative results for the three established protocols are consistent with the best performances of the reported systems. The results for measurements on blood-lipid phantoms are close to the reference values obtained with OxiplexTS and co-oximetry, except a discrepancy was found for the lowest concentration of Intralipid. Venous and arterial cuff occlusions on the forearm resulted in the expected hemoglobin changes that are consistent with the physiology of such hemodynamic challenges. The presented quantitative parameters can be compared to other systems that measured similar tests.

## Data Availability

Data underlying the results presented in this paper are not publicly available at this time but may be obtained from the authors upon reasonable request.
